# Enterprise innovation and audit pricing: An evidence study from China’s A-share listed companies

**DOI:** 10.1371/journal.pone.0300137

**Published:** 2024-03-11

**Authors:** Li Zhang, Wunhong Su, Shanqiu Liu

**Affiliations:** 1 School of Accounting, Guangzhou Huashang College, Guangzhou, China; 2 International College, Krirk University, Bangkok, Thailand; 3 School of Accounting, Hangzhou Dianzi University, Hangzhou, China; Privatuniversität Schloss Seeburg: Privatuniversitat Schloss Seeburg, AUSTRIA

## Abstract

Driven by innovation strategy, Chinese enterprises’ innovation investment, and research and development capability have been continuously improved, and the audit risk caused by this has attracted widespread attention from the academic community. This study takes China’s A-share listed companies from 2013 to 2021 as samples to empirically test the relationship between innovation input and audit pricing of Chinese enterprises. Research shows that the higher the innovation investment, the higher the audit cost. High-quality corporate governance, sufficient research and development personnel, research and development subsidies, and operating cash flow can all play a negative moderating role. A good innovation environment will weaken the positive influence between innovation input and audit fees. This study theoretically confirms the risk-oriented audit pricing mechanism, which is of great significance for optimizing enterprise innovation risk management and improving audit service levels.

## 1 Introduction

As governments around the world advocate the strategic development plan of “mass innovation” and uphold the concept of innovative and high-quality development, the global wave of implementation of innovation has been set off. China, as the world’s largest new economy, is the largest contributor to global R&D spending. The Chinese government actively encourages enterprises to carry out innovative activities so that made in China creates a transformation in China, and more and more Chinese enterprises join the innovation activities. In this context, enterprise innovation has gradually become the focus of scholars. In the broad sense, enterprise innovation mainly refers to the innovation of product design, production, company system, and process in order to maintain a competitive position, maintain the irreplaceability of enterprise products, and conduct scientific and efficient management of enterprises. The enterprise innovation studied in this study mainly refers to the technological innovation of enterprises. By improving the production process and developing new products, enterprises can maintain a degree of product differentiation in the market, obtain a higher premium, and enhance their competitiveness. Innovation is one of the key mechanisms to maintain and promote long-term value growth of enterprises [1].

However, based on the risk-oriented theory, the focus of auditor practice is to assess the risks of the audited entity’s financial statements. The quality of financial reporting is closely linked to audit quality. This connection has deepened scholarly focus on audit quality and driven research into audit fees.In past research, audit fees have garnered significant attention. The determinants of audit fees are broadly categorized into client attributes, auditor attributes, and audit engagement characteristics [2]. The client attributes have the most substantial impact on determining audit fees, primarily including factors such as size, complexity, reputation, inherent risk, profitability, leverage, ownership structure, internal control, governance, and industry [3, 4].The high risk of innovation, the long cycle time, and the volatility of returns are all important factors for auditors to charge premium fees. The uncertainty of innovative activities also enhances the motivation of management to manipulate financial statements and earnings management, increasing audit risks [5, 6]. In order to cope with audit risks brought about by innovative activities, auditors must invest more work to ensure audit quality, so audit fees should be increased. Academic circles have been paying attention to the relationship between innovation input and audit costs. Enterprises can reduce the risk of research and development and reduce the risk premium of audit fees by reducing and eliminating the uncertainty of research and development. At the same time, the regulatory authorities are also paying attention to and controlling the economic risks brought about by innovative activities to improve the effectiveness of the audit market.

This study analyzes the impact of innovation on audit pricing from the perspective of audit risk and analyzes the effect of innovation on audit pricing from the perspective of different innovation environments. Existing studies have paid little attention to the relationship between innovation environment and innovation. This study puts enterprise innovation, innovation environment, and audit fees into a research framework and constructs the logic of “subject—environment—result”. The regulation effect of different internal and external environmental factors on innovation is analyzed. At the same time, based on the different characteristics of enterprises, further discuss the differences in the results of different natures of enterprises so as to facilitate more accurate policy and institutional guidance. The innovation points of this study are as follows: First, it enriched the research on the influencing factors of audit fees. There is existing literature to analyze and study the difference analysis of innovation, audit fee risk premium, and environmental regulation of different types of enterprises from the perspective of firm heterogeneity. Secondly, from the perspective of the innovation environment, it analyzes the mechanism of innovation affecting audit fees and clarifies the regulatory role of the innovation environment. Suppose enterprises want to obtain more external benefits from innovation. In that case, they should pay attention to the role of the innovation environment in innovation, give play to corporate governance, government subsidies, and other means, and better promote innovation while reducing risks and ensuring audit quality to obtain additional benefits. Thirdly, research on innovation issues provides an effective implementation path for enterprises to enhance innovation ability.

The remaining chapters of the study are organized as follows. The second chapter is the literature review, which reviews the relevant literature related to enterprise innovation, audit fees, and innovation environment. The third chapter is the theoretical analysis and research hypothesis, based on the theoretical analysis of the study research hypothesis. The fourth chapter is the research design, introducing the setting of variables and models and the source of data. The fifth chapter is empirical analysis, the empirical analysis of the data, and the robustness test. Chapter six is the conclusion of this study.

## 2 Literature review

### 2.1 Enterprise innovation

In the broad sense, enterprise innovation mainly refers to the innovation of product design, production, company system, and process. In order to maintain the competitive position, maintain the irreplaceability of enterprise products, and conduct scientific and efficient management of enterprises. The enterprise innovation studied in this study mainly refers to the technological innovation of enterprises. By improving the production process and developing new products, enterprises can maintain a degree of product differentiation in the market, obtain a higher premium, and enhance their competitiveness. Innovation is one of the key mechanisms to maintain and promote long-term value growth of enterprises [1, 7].

The influencing factors of enterprise innovation are mainly divided into internal and external factors. In the internal influencing factors of innovation, the personal characteristics of managers and corporate governance occupy the main positions. In terms of personal characteristics of managers, such backgrounds as the length of tenure of senior executives [8], risk preference level of management, the cross-border experience of senior executives [9], and military experience [10] are helpful to promote enterprise innovation. In terms of corporate governance, employee stock ownership plans are conducive to the innovation output of enterprises [11], but some scholars believe that high-intensity equity incentive plans are not conducive to the development of R&D investment activities of enterprises [12]. Due to the high concentration of ownership and managers’ aversion to risk, small private enterprises may lack the willingness to participate in innovation [13]. However, in recent years, China has found in its research on the innovation of enterprises in strategic emerging industries that high ownership concentration can promote enterprise innovation to a certain extent [14]. The research on external factors of innovation mainly focuses on macro policies, intellectual property protection, and public opinion. In terms of macro policies, the research believes that government subsidies are conducive to improving the quantity of enterprise innovation. However, full subsidies before innovation output will cause enterprises to “prioritize quality over quantity” in innovation, but subsidies after innovation output will effectively reduce this phenomenon [15]. compared the promotion effects of tax incentives and subsidy policies on enterprise innovation and found that compared with tax incentives, government subsidies are more conducive to stimulating enterprise innovation. However, both have a certain threshold and when the policy exceeds a certain threshold, it will have a negative effect on innovation, so the two kinds of policies should be used together [16]. The research on investors and media attention found that public opinion pressure can significantly promote enterprises’ green innovation [17].

### 2.2 Audit fees

Studies related to audit fees originated in 1980, when Simunic proposed the audit pricing model, believing that audit pricing was composed of audit cost and audit risk premium, which was in line with the concept of risk-oriented audit [18]. Simultaneously, Simunic [18] noted that auditors with higher market levels could charge an audit premium, levying higher fees than other auditors. This “fee premium” is based on the notion that larger audit firms represent better audit quality.Later, scholar Houston further modified this model and divided audit risk into litigation risk and non-litigation risk. Later studies on audit pricing were mostly carried out based on this model. In contrast to developed countries, China, as an emerging economy, lacks stringent regulatory requirements and demand for audit services. The characteristic of the audit market in emerging economies is the presence of many small audit firms [19], increasing market competition [20]. A distinguishing feature of the corporate sector in emerging markets is a highly concentrated ownership pattern (e.g., family-owned or controlled by major shareholders), which could influence the demand for audit services, thereby affecting audit fees [21].

China’s marketization started relatively late, and China’s study on audit fees was relatively late. Since the China Securities Regulatory Commission (CSRC) introduced the policy of forcing listed companies to disclose audit fees in 2001, most research perspectives have focused on the influencing factors of audit fees and the impact of new financial information disclosure requirements on audit pricing.Existing studies have focused on the influencing factors of audit fees, focusing on the size of the company, the effectiveness of internal control, the nature of the firm, the quality of information disclosure, and corporate governance [2]. In terms of company size, most studies believe that company size is directly proportional to audit fees. Compared with economically developed areas and economically backward areas, enterprise scale is more sensitive to the level of audit fees [22]. Ding found a positive correlation between the size of outstanding shares and audit fees [23]. At the same time, corporate governance has a certain relationship with audit fees. Based on the study on the relationship between Nankai Company’s comprehensive governance index and audit pricing, it is found that the higher the corporate governance index, the lower the audit fees [24]. There is also a significant positive correlation between the effectiveness of internal control and audit fees [25]. It shows that enterprises with good corporate governance face relatively low audit risks, and audit fees are negatively correlated with the corporate governance index. From the perspective of the agency, when there is no separation of two rights and the proportion of major shareholders is low, the proportion of major shareholders is negatively correlated with audit fees.

In contrast, when the proportion of major shareholders is high, the proportion of major shareholders is positively correlated with audit fees. In the case of a higher degree of separation of the two rights, the higher the shareholding ratio of major shareholders, the negative correlation between the shareholding ratio of shareholders and audit pricing is shown [26]. Innovate a high-risk investment activity with high risk, long cycle, and unstable returns [27]. Innovation can bring high profits but also face huge losses from failure. When auditors recognize this risk, they will increase audit investment to ensure audit quality. That is, enterprise innovation input will increase audit costs.

### 2.3 Innovation environment

The European Regional Economic School first proposed the concept of an innovation environment. According to the view of the European Innovation Environment Research Group, enterprises are not independent individuals but products of the environment, and innovative enterprises are also born in the innovation environment. At the same time, there is a mutual influence and interdependence between enterprises and the environment [28, 29]. Chinese scholars believe that the regional innovation environment consists of four levels, namely, basic level network, cultural level network, organizational level network, and information level network. Building a good regional innovation environment requires bottom-up policy guidance or exchanges and cooperation among enterprises, while enterprise innovation will further promote the development of an innovation environment [30]. According to the Chinese Entrepreneur Survey System, China has entered a period of continuous innovation since 2016. However, the innovation environment of enterprises still needs to be further improved in terms of the institutional environment, cultural environment, market environment, and talent environment. In recent years, Chinese scholars have mainly focused on the impact of the innovation environment on enterprise innovation. Through the study of different innovation environment factors and regions, most scholars believe that the innovation environment generally has a positive impact on the innovation ability and innovation output of enterprises. According to the research on the factors affecting the innovation of Chinese industrial enterprises, the innovation environment will significantly increase the innovation behavior of enterprises so as to win more survival opportunities for enterprises. Further research shows that the talent environment in the innovation environment has a significant impact on the innovation ability of enterprises [28].

Corporate governance is an important factor affecting the innovation environment, and the study on the influencing factors of audit pricing enhances auditors’ attention to innovation in the overall innovation environment. This study studies the relationship between innovation activities and audit pricing from the perspective of the innovation environment and discusses the influencing mechanism of innovation separately by distinguishing different internal and external influencing factors of innovation.

## 3 Theoretical analysis and hypothesis

### 3.1 Enterprise innovation and audit fees

In risk-oriented audit, audit pricing is usually related to audit risk. In the face of enterprises with high audit risk, accounting firms need to pay more time and human costs to check errors and prevent fraud, so audit risk constitutes an audit premium. The research holds that when the managerial level is more powerful, and there is a motivation for fraud, enterprise technological innovation will become a means for managers to realize their interests, providing opportunities for fraud and thus increasing audit risk [31]. However, Chinese enterprises have problems with the “patent bubble” and “false innovation” [32], resulting in high innovation costs but low actual innovation output and low conversion rates. High R&D of enterprises brings uncertainty in the valuation and evaluation of intangible assets, which brings greater litigation risk to auditors. As the proportion of intangible assets increases, potential infringement of patents and Copyrights can lead to lengthy litigation for auditors, and auditors with a higher proportion of intangible assets have a higher litigation risk [33]. Innovation leads to higher financial risks, which will increase the motivation of enterprises to commit fraud. However, the high R&D investment of enterprises is often an expression of the implementation of an offensive corporate strategy, and such companies will adopt more aggressive financing models to reduce the robustness of accounting and further aggravate the audit risk [34]. Therefore, innovative enterprises often face higher operational risks, which intensifies audit risks and increases audit costs. Based on the above analysis, this study proposes hypothesis 1.

Hypothesis 1: There is a positive correlation between enterprise innovation and audit fees.

### 3.2 The regulatory role of the innovation environment

On the spatial dimension, the innovation environment is currently divided into the national level, the regional level, and the enterprise level. In terms of connotation, the innovation environment is mainly divided into a hard power environment based on material conditions and a soft power environment based on system, policy, and quality of workers. It is found that R&D investment intensity is positively related to audit costs, and the audit risk of high R&D enterprises is lower than that of low R&D enterprises. There is a clear difference in the R&D environment to this effect [35]. Tax subsidies can increase enterprises’ net cash flow and improve the efficiency of their innovation output [36]. By means of tax incentives and subsidies, the government encourages independent research and development of enterprises and reduces the burden of enterprise research and development. Tax subsidies have a good information transmission effect, and the positive signal to the outside world can absorb more social capital [37, 38]. In the soft environment of the enterprise level, corporate governance environment, intellectual property innovation policy, and talent quality constitute the main components of the enterprise innovation environment. Among enterprises in the cultural industry, studies have confirmed that a good corporate governance environment and internal control can effectively improve the innovation performance of enterprises [39]. In addition, enterprise innovation investment requires enterprises to have abundant cash flow and good material conditions. Among semes, good corporate governance mechanisms and internal control environments are the main elements of an innovation environment, which can reduce audit risks and, thus, audit fees [40]. With the size of assets, the quick ratio of enterprises is negatively correlated with audit fees. Based on the above analysis, hypothesis 2 is proposed in this study.

Hypothesis 2: The innovation environment plays a moderating role in the correlation between innovation and audit fees. The better the innovation environment, the weaker the positive correlation between innovation and audit fees

## 4 Research design

### 4.1 Sample selection and data sources

This study selects the sample data of China’s A-share listed companies from 2013 to 2021, excludes ST, financial, and incomplete enterprise data, and removes the samples with zero innovation input. Meanwhile, in order to prevent the impact of extreme values on the sample results, Winsor is used to indent the variable samples in the regression model at 1% and 99% quantiles. Finally, 12006 valid samples were obtained. All data in this study were collected from SCAMAR and WIND databases. Data processing was summarized through EXCEL2010, and empirical analysis was performed using Stata15.1.

### 4.2 Variable definition and model construction

In order to test whether hypothesis 1 is true, the following regression model is constructed with reference to the research of Zhang Rui and Wang Yangyang [34] to verify the relationship between R&D input and audit fees. If α1 is significantly positive, it indicates that the R&D input of enterprises will increase the audit fees of auditors.


$$Lnfee_{i,t}=\alpha_{0}+\alpha_{1}\;RD_{i,t}+\sum \alpha_{j}\;controls_{i,t}+\sum Year+\varepsilon_{i,t}$$ (1)

In model 1, the audit fee (Lnfee) is the explained variable, which is different from that of existing scholars. He Qin et al. adopted the same measurement method, choosing the natural logarithm of audit fee to represent the level of audit fee [41]. Research and development investment (R&D) is an explanatory variable representing an enterprise’s degree of innovation. Referring to the research literature of Liu et al. [42], the logarithm of R&D investment is used to represent the level of innovation investment of an enterprise. Meanwhile, the influence factor of enterprise size is considered in the robustness test. Two relative value indicators, the ratio of R&D investment to total assets (RA) and the ratio of R&D investment to operating income (RI), were used to replace the test. The influence of absolute value and relative value is considered comprehensively here, which makes the research results more robust. An important factor affecting the choice of client and auditor is the audit fee. Existing studies have found that factors affecting audit fees include customer scale, listing years, asset-liability ratio, business complexity, risk status, and profitability, all of which have a significant impact on audit fees [43, 44]. Therefore, in terms of the selection of control variables, we refer to the studies of existing scholars [41, 43] and consider the impact on audit fees from different perspectives, such as the enterprise’s own operation and business complexity. Select company Size (Size), asset-liability ratio (Lev), current ratio (Rec), inventory ratio (Inv), main business revenue Growth rate (Growth), return on assets (Roa), whether Loss occurred (Loss), firm size (Big4), and last year’s audit opinion type (Lop), while controlling for year (Year) annual fixed effects. The specific variable definitions and meanings are shown in Table 1.

**Table 1:** Variable definitions.

Variable type	Variable name	Variable symbol	Variable definition
Explained variable	Audit fee	lnfee	The natural logarithm of the current audit fee
explain variable	Innovation input	R D	R&D investment
		RA	Proportion of R&D investment/total assets
		R I	R&D investment/revenue ratio
Moderating variables	Corporate governance environment	Cgn	The first principal component obtained in the principal component analysis is used as a comprehensive index to reflect the level of corporate governance.
	The proportion of R&D personnel	Person	The proportion of the number of R&D personnel, the ratio of the number of R&D personnel to the total number of employees
	Research and development subsidy	subsidy	The amount of research and development grants for the current year is logarithmic.
	Operating cash flow	Ocf	Net cash flow from operating activities for the year divided by total assets at the end of the period
Controls	Variable Company	Size	The natural logarithm of total revenue for the period
	Asset-liability ratio	Lev	Total ending liabilities/total ending assets
	Current ratio	Current	assets/current liabilities
	Accounts receivable ratio	Rec	Ending accounts receivable/total ending assets
	Inventory ratio	Inv	Total ending inventory/ending assets
	Growth rate of main business income	Growth	current year’s main business income—last year’s main business income)/last year’s main business income
	Return on assets	Roa	Net profit for the period/total assets at the end of the period
	The value of firm size	Big4	Big4 is 1; otherwise, it is 0
	Previous year’s Audit Opinion Type	Lop	The previous year’s annual report was issued with a non-unqualified opinion with a value of 1; otherwise, 0
	Annual variable	Year	control

In order to test whether hypothesis 2 is valid, model (2) is set to verify the moderating role of the R&D environment in the relationship between R&D input and audit fees. In the formula, X represents the adjustment factor variables of the innovation environment that are different in Cgn, Person, R&D subsidy, and operating cash flow (Ocf), while year represents annual effect. If β_1_, β_2_, and β_3_ are significant, it indicates that the regulating variables play a moderating role.


$$Lnfee_{i,t}=\beta_{0}+\beta_{1}\;RD_{i,t}+\beta_{2}\;X_{i,t}+\beta_{3}\;RD_{i,t}\times X_{i,t}+\sum \beta_{j}controls_{i,t}+\sum Year+\delta_{i,t}$$ (2)

To examine the effect of the innovation environment on innovation, the moderating variables of the innovation environment were selected: Corporate governance environment (Cgn), ratio of R&D personnel (Person), R&D subsidy (Ocf), and operating cash flow (OCF). For the reference to the corporate governance environment [45], the first principal component obtained from the principal component analysis method is adopted as the comprehensive index reflecting the level of corporate governance. The principal component analysis method constructs comprehensive indicators from the aspects of supervision, incentive, and decision-making to measure the level of corporate governance. The incentive mechanism in corporate governance is represented by executive compensation and executive shareholding ratio; the supervisory role of the board of directors is represented by the proportion of independent directors and the scale of the board of directors; and the supervisory role of the ownership structure is represented by the proportion of institutional shareholding and the degree of equity balance (the sum of the shareholding ratio of the second to the top five shareholders/the shareholding ratio of the controlling shareholders). The decision power of the general manager is represented by seven indicators of whether the chairman and the general manager are in combination, and the corporate governance index is constructed by principal component analysis. The first principal component obtained in the principal component analysis is used as a comprehensive index reflecting the level of corporate governance, and the higher the score, the better the level of governance. Executives are defined in a broad sense, including directors, supervisors, and senior management.

## 5 Empirical analysis

### 5.1 Descriptive statistics

Table 2 shows the results of descriptive statistical analysis. According to the results, it can be found that the mean value of audit fees is 13.676, the standard deviation is 0.684, the maximum value is 17.977, and the minimum value is 11.918, indicating that the sample difference is not too large, and the results are basically consistent with the existing research [46]. The maximum value of innovation input is 10.34, the minimum value is 4.273, the mean value is 7.801, and the median value is 7.790, indicating that the whole sample conforms to the normal distribution. From the sample governance level, it can be seen that the maximum value is 3.308, the minimum value is -3.020, and the variance is 1, indicating that the governance level of sample enterprises fluctuates greatly. The governance levels of different enterprises are uneven and different. From the statistical data of the proportion of R&D personnel and R&D subsidies, the sample difference is also relatively large, which provides a good research basis for subsequent research. The mean value of whether the firm is a “Big Four” is 0.051, indicating that 5.1% of listed companies choose the Big Four accounting firms for auditing. The reasons for the low proportion of the four major companies are mainly high audit costs, and the customer groups targeted by the four major companies are mainly large group companies. At the same time, the quality of small and medium-sized enterprises in the Chinese market is uneven, and the audit risk is high, which also makes the four major companies reluctant to undertake. Descriptive statistical analysis results and existing studies [46, 47] are basically consistent, which confirms the accuracy of the analysis results in this study.

**Table 2:** Descriptive statistics.

variable	N	mean	sd	min	p50	max
lnfee	12006	13.676	0.684	11.918	13.592	17.977
RD	12006	7.801	0.634	4.273	7.790	10.340
Cgn	12006	-0.007	1.018	-3.020	-0.124	3.308
Person	12006	0.174	0.154	0	0.131	1.405
Ocf	12006	0.046	0.067	-0.463	0.043	0.661
Subsidy	12006	7.087	0.862	0	7.117	9.762
Size	12006	21.585	1.428	16.431	21.437	28.554
Lev	12006	0.407	0.199	0.008	0.399	1.698
Current	12006	2.662	4.012	0.094	1.697	144.00
Rec	12006	0.135	0.101	0	0.117	0.701
Growth	12006	0.152	0.732	-0.913	0.098	55.044
Inv	12006	0.137	0.104	0	0.116	0.861
Roa	12006	0.034	0.074	-1.648	0.034	0.526
Big4	12006	0.051	0.219	0	0	1
Lop	12006	0.005	0.073	0	0	1

### 5.2 Univariate correlation analysis

Table 3 shows the correlation analysis among single variables. It can be seen from the results that there is a significant positive correlation between the explanatory variable R&D input and audit fees, which is consistent with the hypothesis which provides certain supporting evidence for the research. There were significant correlations among the corporate governance environment (Cgn), R&D staff ratio (Person), R&D subsidy (Ocf), explanatory variable R&D investment, and explanatory variable audit fees. There is also a significant correlation between most control variables and audit fees, which is generally consistent with the expectation which provides a good basis for subsequent research. In addition, the multicollinearity test among variables is also carried out, and the variance expansion factor is all below 2, indicating that there is no multicollinearity between variables.

**Table 3:** Descriptive statistics.

	lnfee	RD	Cgn	Person	Ocf	Subsidy	Size	Lev	Current	Rec	Growth	Inv	Roa	Big4	Lop
lnfee	1														
RD	0.499***	1													
Cgn	-0.287***	-0.229***	1												
Person	-0.152***	0.126***	0.204***	1											
Ocf	0.072***	0.111***	-0.094***	-0.077***	1										
Subsidy	0.439***	0.458***	-0.234***	-0.078***	0.068***	1									
Size	0.705***	0.615***	-0.488***	-0.256***	0.15***	0.503***	1								
Lev	0.375***	0.232***	-0.323***	-0.208***	-0.135***	0.262***	0.541***	1							
Current	-0.218***	-0.14***	0.213***	0.213***	0.02	-0.163***	-0.328***	-0.488***	1						
Rec	-0.07***	0.142***	0.136***	0.22***	-0.212***	-0.021	-0.063***	0.09***	-0.069***	1					
Growth	0.018	0.03	0.045***	0.042***	-0.01	0.006	0.015	-0.007	-0.019	0.03	1				
Inv	0.023	-0.021	-0.08***	-0.11***	-0.158***	0.004	0.124***	0.269***	-0.112***	-0.021	-0.03	1			
Roa	-0.039***	0.099***	-0.032	0.058***	0.342***	0.019	0.079***	-0.297*	0.114***	-0.043***	0.092***	-0.048***	1		
Big4	0.444***	0.255***	-0.185***	-0.048***	0.076***	0.208***	0.365***	0.1372***	-0.071***	-0.074***	-0.014	-0.006	0.038***	1	
Lop	0.017	-0.04***	0.012	-0.01	-0.024	-0.016	-0.027	0.049*	-0.019	0.004	-0.021	-0.005	-0.104*	-0.007	1

Note

***, **, * indicate significance at 1%, 5%, 10% levels, respectively. Values in parentheses are t-values.

### 5.3 Regression results of innovation input and audit fees

In order to verify whether hypothesis 1 is valid, the regression analysis results are shown in Table 4 (1). The regression results are consistent with hypothesis 1, and there is a significant positive correlation between R&D input (RD) and audit fees. It shows that based on the consideration of risk control, auditors will realize the increase of audit input and audit risk brought by R&D, as well as the potential motivation of earnings management and the risk of fraud and misstatement of enterprises, and charge risk premium to increase audit fees. Company Size (Size), Current ratio (Current), main business revenue Growth rate (Growth), firm size (Big4), last year’s audit opinion type (Lopinion), accounts receivable ratio (Rec), inventory ratio (Inv) and audit fees are significantly positively correlated, indicating that the larger the company’s operation scale, The more complex the business, the higher the audit risk, and the larger the firm size, the higher the audit fee. Return on assets (Roa) has a significant negative correlation with audit fees and is consistent with the expected results.

**Table 4:** The moderating effects of basic regression and innovation environment.

variable	(1)	(2)	(3)	(4)	(5)
	Fundamental regression	Corporate governance environment	Proportion of R&D personnel	Research and development subsidy	Operating cash flow
RD	0.082*** (9.52)	0.070*** (7.75)	0.086** (7.95)	0.438*** (9.91)	0.091*** (9.56)
Cgn		0.216*** (4.12)			
RD*Cgn		-0.022*** (-3.23)			
Person			0.941*** (2.62)		
RD*Person			-0.107** (-2.36)		
Subsidy				0.459*** (10.14)	
RD*Subsidy				-0.069*** (-11.56)	
Ocf					1.062* (1.52)
RD*Ocf					-0.181** (-1.99)
Size	0.27*** (57.07)	0.286*** (57.78)	0.276** (55.24)	0.251*** (51.99)	0.272*** (57.32)
Lev	0.021 (0.69)	0.045 (1.49)	0.017 (0.57)	0.008 (0.28)	0.007 (0.22)
Current	0.003*** (2.75)	0.003** (2.16)	0.003** (2.21)	0.003 (2.20)	0.003** (2.53)
Rec	0.246*** (5.79)	0.295*** (6.92)	0.266*** (6.19)	0.212*** (5.04)	0.288*** (6.67)
Growth	0.014** (2.55)	0.011** (2.01)	0.014** (2.41)	0.013 (2.40)	0.013** (2.39)
Inv	0.264*** (6.40)	0.268** (6.54)	0.254*** (6.17)	0.241*** (5.90)	0.294** (7.07)
Roa	-0.808*** (-11.08)	-0.77*** (-10.59)	-0.808*** (-11.07)	-0.775*** (-10.73)	-0.704*** (-9.29)
Big4	0.679*** (33.81)	0.674*** (33.53)	0.676*** (33.62)	0.632*** (31.22)	0.681*** (33.88)
Lopinion	0.227*** (4.07)	0.226*** (4.06)	0.229*** (4.11)	0.229*** (4.14)	0.232*** (4.17)
Constant	7.107*** (88.28)	6.857*** (79.52)	6.927*** (69.21)	0.251*** (31.71)	7.011*** (81.25)
Year	Yes	Yes	Yes	Yes	Yes
F	906.236	828.858	817.300	845.151	818.629
Adj-R2	57.6%	58%	57.7%	58.5%	57.7%
N	12006	12006	12006	12006	12006

Note

***, **, * indicate significance at 1%, 5%, 10% levels, respectively. Values in parentheses are t-values.

### 5.4 The regulatory role of the innovation environment

The innovation environment will affect the effect of innovation implementation and the effectiveness of transformation into economic results. Whether auditors can identify the impact of innovation environment factors on innovation and reduce the risk compensation of charging high audit fees in the audit process, this study further tests the regulatory effect of the innovation environment. The empirical test results are shown in Table 4 (2)-(5). The results showed that the test results of the four moderating variables of innovation environment: Corporate governance environment (Cgn), Person, R&D subsidy, and operating cash flow (Ocf) were significant, suggesting that all of them play a moderating role in varying degrees, supporting the original hypothesis. Through the incentive mechanism of executive compensation and executive shareholding ratio, the supervision mechanism of independent directors and directors, the check and balance mechanism of institutional shareholders and equity checks, and the dual role of chairman and general manager—enhancing decision-making power, enterprises improve the corporate governance environment from various aspects of supervision, incentive, and decision-making. Auditors reduce the premium on audit fees due to R&D investment by identifying a good governance environment. At the same time, auditors can also identify that the ratio of R&D personnel and R&D subsidy in the enterprise provides R&D protection to the sufficient cash flow of the enterprise, reducing the risk of R&D investment. The original hypothesis 2 adjustment mechanism pathway has also been verified.

### 5.5 Robustness test

In order to solve the robustness problem of the model, this study attempts to replace the absolute variable of the logarithm of the explanatory variable RD R&D investment with two relative variables, RA R&D investment to total assets and RI R&D investment to business income, for the robustness test. The robustness test results of basic regression are shown in Table 5, which still support the research conclusion that there is a positive correlation between R&D input and audit fees. Meanwhile, a robustness test is also conducted for the regulatory effect, and the results are shown in Tables 6 and 7. The test results of four adjustment effects of corporate governance environment (Cgn), R&D personnel ratio (Person), R&D subsidy (Ocf), and operating cash flow (OCF) are still significant, which supports the previous conclusion, and the conclusion is robust. This study also tried to distinguish two sub-samples with different levels according to the median R&D investment for regression test, reduced the sample by 20% for regression analysis, and found that the results were still robust.

**Table 5:** Robustness test of basic regression.

variable	(1)	(2)
RA	2.912***(13.03)	
RI		0.905***(11.05)
Cgn	0.05***(10.79)	0.048***(10.47)
Person	0.309***(9.82)	0.057*(1.82)
Ocf	0.269***(3.93)	0.3***(4.42)
Subsidy	0.073***(13.33)	0.06***(10.93)
Controls	Yes	Yes
Year	Yes	Yes
Constant	6.404***(75.68)	6.979***(84.78)
F	803.843	910.351
Adj-R2	60.7%	57.8%
N	12006	12006

Note

***, **, * indicate significance at 1%, 5%, 10% levels, respectively. Values in parentheses are t-values.

**Table 6:** Test of the robustness of regulation 1.

variable	Corporate governance environment	Proportion of R&D personnel	Research and development subsidy	Operating cash flow
RA	1.464**(6.98)	3.893**(10.73)	5.68***(4.03)	1.753**(6.58)
Cgn	0.041**(6.25)			
RD*Cgn	-0.373**(-2.01)			
Person		0.157***(3.75)		
RD*Person		-4.845*** (-5.42)		
Subsidy			0.094*(13.97)	
RD*Subsidy			-1.04**(-5.33)	
Ocf				0.4***(4.48)
RD*Ocf				-5.966** (-2.28)
Constant	6.919*** (81.43)	7.118*** (87.09)	7.042** (83.79)	7.201*** (89.64)
Year	Yes	Yes	Yes	Yes
F	824.736	825.393	834.865	813.006
Adj-R2	57.9%	57.9%	58.2%	57.6%
N	12006	12006	12006	12006

Note

***, **, * indicate significance at 1%, 5%, 10% levels, respectively. Values in parentheses are t-values.

**Table 7:** Test of the robustness of regulation effect II.

variable	Corporate governance environment	Proportion of R&D personnel	Research and development subsidy	Operating cash flow
RI	0.850**(7.75)	0.086*** (7.95)	5.955**(10.02)	0.911***(9.56)
Cgn	0.042**(7.01)			
RD*Cgn	-0.130*(-1.66)			
Person		0.941**(2.62)		
RD*Person		-0.107* (-2.36)		
Subsidy			0.093*** (10.02)	
RD*Subsidy			-0.734*** (-8.83)	
Ocf				0.27***(3.45)
RD*Ocf				-0.766(-0.79)
Constant	6.688* (76.65)	6.927** (69.21)	6.782** (80.19)	6.952** (84.14)
Year	Yes	Yes	Yes	Yes
F	832.476	817.300	842.636	821.567
Adj-R2	58.1%	57.7%	58.4%	57.8%
N	12006	12006	12006	12006

Note

***, **, * indicate significance at 1%, 5%, 10% levels, respectively. Values in parentheses are t-values.

### 5.6 Heterogeneity analysis

Yin et al. [48] researched different types of technology, capital, and labor-intensive enterprises. They found that innovation input did not affect the performance of labor-intensive enterprises but had a significant impact on the performance of technology and capital-intensive enterprises. Technology-intensive enterprises had a changing trend of first increasing and then decreasing. It can be seen that enterprises of different natures have different responses to R&D investment due to their differences in financing, strategic development, growth, and sensitivity to the R&D environment. Based on the research [47], the samples were grouped according to the nature of equity ownership to test the effect of difference of firm heterogeneity on audit fees in an innovation environment and further analyze the influence mechanism of the difference of R&D environment on the model adjustment effect.

Table 8 Regression results show the difference between state-owned enterprises and non-state-owned enterprises in the adjustment effect caused by firm heterogeneity according to the sample group regression test. Corporate governance of both state-owned enterprises and non-state-owned enterprises still has a significant negative moderating effect on the impact of R&D input on audit fees, indicating that strengthening corporate governance can significantly inhibit audit risk premiums for both state-owned enterprises and non-state-owned enterprises.

**Table 8:** Results of heterogeneity test.

variable	Corporate governance environment		Proportion of R&D personnel		Research and development subsidy		Operating cash flow	
	state-owned	non-state	state-owned	non-state	state-owned	non-state	state-owned	non-state
RD	0.091*** (5.46)	0.083 *** (7.33)	0.089 *** (5.25)	0.06 *** (4.17)	0.553 *** (8.20)	0.134 ** (-2.18)	0.072 *** (4.77)	0.094 *** (7.64)
Cgn	-0.186 *** (-1.79)	0.337 *** (5.19)						
RD*Cgn	0.033 *** (2.49)	-0.042 *** (-5.10)						
Person			1.659 *** (2.67)	-0.26 (-0.61)				
RD*Person			-0.203*** (-2.61)	0.047 (0.88)				
Subsidy					0.536 (7.69)	0.175 *** (-2.78)		
RD*Subsidy					-0.081 (-9.05)	-0.028*** (3.35)		
Ocf							0.16 (0.14)	1.732 ** (2.04)
RD*Ocf							-0.08 (-0.53)	-0.26** (-2.35)
Control	Yes							
Year	Yes							
Constant	5.546 *** (35.24)	7.705 *** (73.20)	5.694 *** (33.98)	7.795 *** (61.99)	10.63 *** (19.67)	9.36 *** (19.63)	5.899 *** (40.28)	7.649 (70.65)
F	371.777	429.964	366.070	428.291	389.395	430.727	366.636	70.65
Adj-R2	63.1%	53%	62.8%	52.9%	64.2%	53.1%	62.8%	52.9%
N	4362	7644	4362	7644	4362	7644	4362	7644

Note

***, **, * indicate significance at 1%, 5%, 10% levels, respectively. Values in parentheses are t-values.

Based on the advantages of financing and obtaining funds, state-owned enterprises have stronger innovation ability and are more likely to participate in some large-scale R&D projects with long R&D cycles and large R&D investment. Hence, they need to invest more in R&D personnel to ensure the smooth progress of R&D. Meanwhile, state-owned enterprises are less flexible in personnel management than non-state-owned enterprises, so the innovation efficiency of R&D personnel is not advantageous. Therefore, the investment of R&D personnel can stimulate and guarantee the success rate of R&D transformation results and reduce the risks brought by R&D. The results of empirical analysis have also confirmed that R&D personnel play a significant role in the regulation of state-owned enterprises.

State-owned enterprises are more likely to get government subsidies than private enterprises, and only politically connected enterprises in private enterprises are more likely to get government subsidies [49]. The thought of “state for the people” of state-owned enterprises has a certain policy inclination in the policy, and the pressure of capital is small, and the difficulty and cost of obtaining financing preferential treatment are lower than that of non-state-owned enterprises in obtaining external financing [50]. Due to the slanting nature of government subsidies, R&D subsidies of state-owned enterprises are relatively easier to obtain than those of non-state-owned enterprises. Government subsidies can also be seen as an effective signal to foreign investors [51]. For non-state firms, auditors are more likely to attribute government subsidies to close government ties and better policy information. At the same time, state-owned enterprises have natural advantages in terms of financing and capital acquisition, so R&D subsidies and operating cash flow have no significant regulating effect on state-owned enterprises. On the contrary, non-state-owned enterprises have great competitive pressure and sufficient motivation for research and development but are restricted by financing constraints. R&D subsidies can provide enterprises with sufficient cash flow, meet the capital needs of enterprises, stimulate more research and development investment and output of non-state-owned enterprises, reduce the research and development risks caused by lack of funds, and weaken the risk premium of auditors’ audit fees.

## 6 Conclusion and prospect

From the perspective of auditors, this study analyzes how Chinese enterprises respond to the national call to carry out innovative activities and how auditors respond to audit fees. Against the background of the continuous growth of Chinese enterprises’ innovation activities, the prevention of audit risks is a problem worth studying. It is found that innovation input is an important factor affecting audit fees. Auditors have an awareness of risk identification, facing the audit risks brought by innovation, by investing more work and increasing audit fees to ensure audit quality. The mechanism test shows that different innovation environments play a significant role in regulation. The innovation environment of the company is also one of the factors that the auditor considers in the audit fee. Both external innovation environment (R&D subsidies) and internal innovation environment (corporate governance environment, number of R&D personnel, operating cash flow) have a significant negative impact on innovation investment. As an effective guarantee for the results of innovation activities, a good innovation environment can significantly reduce the positive correlation between innovation input and audit fees. The reason is that the innovation environment is an effective guarantee for the success of innovation activities, and the transmission of this effective information can reduce the level of auditors’ risk assessment of innovation activities. Therefore, the innovation environment is recognized by auditors as a moderating factor of innovation risk.

Further heterogeneity analysis based on ownership found that there is a significant difference in this effect between state-owned and private enterprises. Compared with non-state-owned enterprises, the innovation environment adjustment effect of R&D personnel in state-owned enterprises is significant. State-owned enterprises can stimulate and guarantee the success rate of R&D transformation results by flexibly mobilizing the investment of R&D personnel and reducing the risks brought by R&D. At the same time, the abundant funds provided by government subsidies and sufficient cash flow are more able to escort the innovation activities of private enterprises lacking financial support and reduce audit risks. Further research provides an effective basis for the more accurate implementation of the policy.

The research results of this study have a certain reference value for regulatory agencies. First, the innovation activities of enterprises continue to increase. While encouraging innovation, the government and relevant regulatory departments should also pay attention to the external economic consequences caused by enterprises’ innovation investment. Since innovation input can significantly increase audit fees, it means that there may be some risks behind enterprise innovation. The regulatory authorities should pay enough attention to and control the risks brought by enterprise innovation input, reduce the earnings manipulation behaviors of enterprises due to performance pressure brought by innovation activities, and thus reduce audit risks and improve the effectiveness of the audit market. In this regard, the government and regulatory authorities should improve the relevant regulatory system in a timely manner and standardize the innovative behavior of enterprises. Second, enterprises should strengthen the construction of self-governance to improve the corporate governance environment, and private enterprises should strengthen the construction of their internal innovation environment to ensure reasonable operating cash flow. State-owned enterprises should rationally match the number of R&D personnel, especially paying attention to management incentives, supervision, and checks and balances. While ensuring that enterprises can obtain market competitiveness through innovation, they can also increase their bargaining ability with auditing firms. Reduce audit fees. Third, while encouraging enterprise innovation, regulators should pay more attention to the innovation environment and create a good innovation environment. Private enterprises should increase research and development subsidies, maintain the legal environment, and improve the system construction at the policy level of the innovation environment while protecting intellectual ownership so as to promote the effective implementation of innovation better. Fourth, innovation environment information has information content. Enterprises need to pay attention to the information disclosure of the innovation environment, actively disclose corporate governance environment, research and development personnel, research and development subsidies, operating cash flow, and other information, transmit favorable signals, reduce innovation risk information known to the market, so as to obtain more government subsidies and investors’ attention and help enterprises gain competitive advantages.
